# *miR‐145* transgenic mice develop cardiopulmonary complications leading to postnatal death

**DOI:** 10.14814/phy2.15013

**Published:** 2021-09-14

**Authors:** Shelby Thomas, Sathiyanarayanan Manivannan, Dwitiya Sawant, Karthik M. Kodigepalli, Vidu Garg, Simon J. Conway, Brenda Lilly

**Affiliations:** ^1^ Center for Cardiovascular Research and The Heart Center Nationwide Children’s Hospital Columbus Ohio USA; ^2^ Department of Pediatrics The Ohio State University Columbus Ohio USA; ^3^ HB Wells Center for Pediatric Research Indiana University School of Medicine Indianapolis Indiana USA; ^4^ Department of Pediatrics Medical College of Wisconsin Milwaukee WI USA

**Keywords:** cardiovascular, *miR‐145*, pulmonary, smooth muscle differentiation

## Abstract

**Background:**

Both downregulation and elevation of microRNA *miR*‐*145* has been linked to an array of cardiopulmonary phenotypes, and a host of studies suggest that it is an important contributor in governing the differentiation of cardiac and vascular smooth muscle cell types.

**Methods and results:**

To better understand the role of elevated *miR*‐*145 in utero* within the cardiopulmonary system, we utilized a transgene to overexpress *miR*‐*145* embryonically in mice and examined the consequences of this lineage‐restricted enhanced expression. Overexpression of *miR*‐*145* has detrimental effects that manifest after birth as overexpressor mice are unable to survive beyond postnatal day 18. The *miR*‐*145* expressing mice exhibit respiratory distress and fail to thrive. Gross analysis revealed an enlarged right ventricle, and pulmonary dysplasia with vascular hypertrophy. Single cell sequencing of RNA derived from lungs of control and *miR*‐*145* transgenic mice demonstrated that *miR*‐*145* overexpression had global effects on the lung with an increase in immune cells and evidence of leukocyte extravasation associated with vascular inflammation.

**Conclusions:**

These data provide novel findings that demonstrate a pathological role for *miR*‐*145* in the cardiopulmonary system that extends beyond its normal function in governing smooth muscle differentiation.

## 1 INTRODUCTION

MicroRNAs *miR*‐*143* and *miR*‐*145* exist in a coexpressed cluster that has been collectively studied and shown to have functions in cancer, stem cell biology, and the cardiovascular system (Boettger et al., 2009; Cheng et al., 2009; Cordes et al., 2009; Jakob & Landmesser, 2012; Vacante et al., 2019; Xin et al., 2009; Ye et al., 2019). Embryonically, *miR*‐*143*/*145* expression is restricted to the cardiovascular system, where they are transcriptionally controlled by myocardin, a smooth muscle and cardiac muscle‐specific master regulator (Cordes et al., 2009; Wang et al., 2001; Xin et al., 2009). Their role in vascular smooth muscle cells is well documented, with several reports demonstrating an ability to govern smooth muscle plasticity, cytoskeletal dynamics, and function to support a differentiated contractile phenotype (Boettger et al., 2009; Cheng et al., 2009; Cordes et al., 2009; Elia et al., 2009; Xin et al., 2009). The actions of *miR*‐*143*/*145* in cardiovascular disease has also been examined. *miR*‐*143*/*miR*‐*145*‐deficient mice exhibit reduced blood pressure and have blunted agonist‐induced contractile responses (Boettger et al., 2009; Xin et al., 2009). In carotid injury and ApoE atherosclerosis models, the expression of *miR*‐*143*/*145* is downregulated, coinciding with a dedifferentiated state (Boettger et al., 2009; Cheng et al., 2009; Cordes et al., 2009; Elia et al., 2009). Further, modulation of *miR*‐*143*/*145* by genetic deletion or viral delivery indicated a role in neointimal formation in various injury models, suggesting a regulatory function in disease‐induced phenotypic modulation (Boettger et al., 2009; Cheng et al., 2009; Cordes et al., 2009; Elia et al., 2009; Vacante et al., 2019; Xin et al., 2009). In the lung, *miR*‐*143*/*145* has been shown to be increased in human patients with pulmonary arterial hypertension (Caruso et al., 2012; Deng et al., 2015). Mouse models of pulmonary hypertension that are deficient in *miR*‐*143*/*145* show decreased disease severity, indicating these microRNAs contribute to disease pathology (Caruso et al., 2012; Deng et al., 2015).

Despite their shared transcriptional control, *miR*‐*143* and *miR*‐*145* have unique targets and govern distinct cellular activities (Vacante et al., 2019). *miR*‐*145* has been shown to uniquely target *Klf4* and *Klf5* (Cheng et al., 2009; Cordes et al., 2009; Xin et al., 2009). Klf4 is a key player not only in stem cell pluripotency, but also in smooth muscle phenotypic switching (Jakob & Landmesser, 2012; Zheng et al., 2010). The downregulation of *Klf4* by *miR*‐*145* is linked to its distinct ability to promote myocardin activation leading to an increase in smooth muscle gene expression (Albinsson et al., 2010; Cordes et al., 2009). Functionally, *miR*‐*145* was shown to independently drive smooth muscle cell fate (Cordes et al., 2009). In other cell types, *miR*‐*145* was reported to promote the differentiation of murine cardiac and lung myofibroblasts (Wang et al., 2014; Yang et al., 2013) and to promote gut epithelium differentiation in zebrafish (Zeng et al., 2009). Thus, *miR*‐*145* appears to play a pivotal role in cell fate and differentiation mechanisms.

Recently, we generated a transgenic mouse that conditionally overexpresses *miR*‐*145* in a Cre recombinase‐dependent manner (Sawant et al., 2020). Here, we characterize the phenotype of these transgenic mice that overexpress *miR*‐*145* in the developing cardiopulmonary system. Our data show that *miR*‐*145* overexpression results in fully penetrant postnatal lethality prior to weaning. These mice have evidence of pulmonary hypertension with muscularization of the pulmonary arteries associated with right ventricular hypertrophy. Single cell RNA‐sequencing revealed that overexpression of *miR*‐*145* in the lungs promotes a pathological phenotype with an increase in innate and adaptive immunity genes and leukocyte adhesion at the vascular wall consistent with tissue inflammation. These data support the notion that *miR*‐*145* contributes to cardiovascular maintenance and its overexpression leads to pathology that is consistent with pulmonary hypertension. Our results indicate that *miR*‐*145* function extends beyond the regulation of smooth muscle differentiation.

## 2 MATERIALS AND METHODS

### 2.1 Mouse lines

The following mouse lines were used in this study: *miR*‐*145* transgenic *(miR*‐*145Tg)* mice produced and characterized by our lab (Sawant et al., 2020), *Myocd*‐*Cre (B6*;*129S6*‐*Myocd^tm1(cre)Jomm^*/*J) mice* (Long et al., 2007) from Jackson Laboratory, and *Rosa26*‐*LacZ (R26R) (Gt(ROSA)26Sor^tm1Sor^) mice* (Soriano, 1999) from Jackson Laboratory. *miR*‐*145Tg* and *R26R* females were bred with *Myocd*‐*Cre* males to generate progeny for analysis. Both male and female mice were used, and we did not observe significance differences in results based on gender at any time points assessed. To genotype, tissue was collected from pups between postnatal days 5–9 and digested with proteinase K. Polymerase chain reaction (PCR) was performed on digested toes using the following primers: miR‐145Tg F: 5’‐ TCC CAC AAC GAG GAC TAC A, miR‐145Tg R: 5’‐GCT AAG CCA TGA CCT CAA GAA, Myocd‐Cre F: 5’‐TGC CAC GAC CAA GTG ACA GC, Myocd‐Cre R: 5’‐CCA GGT TAC GGA TAT AGT TCA, R26R F: 5’ – CGG TGA ATG GTG CTG CGT TGG A. R26R R: 5’ – ACC ACC GCA CGA TAG AGA TTC. Mouse studies were carried out in accordance with the protocols approved by the Institutional Animal Care and Use Committee at the Abigail Wexner Research Institute at Nationwide Children's Hospital.

### 2.2 Fulton index measurement

After flushing with phosphate buffered saline (PBS), hearts were removed, and the right ventricle was separated from the left ventricle and septum. Weights of the right ventricle and the left ventricle with the septum were taken. Fulton Index was calculated as the ratio of the right ventricular weight to the weight of the left ventricle and septum (RV/LV+S).

### 2.3 Histology and immunostaining

Heart and lungs were flushed with PBS. Lungs were inflate‐fixed for 5 min with 4% paraformaldehyde (PFA) then fixed overnight. Organs were embedded in paraffin and sectioned at a thickness of 6 μm. Sections were stained with hematoxylin and eosin (H&E) to visualize morphology and pulmonary vascularization. H&E stained slides were imaged on an Olympus BX51 with a 40x objective, an Olympus SZX7 at 2x magnification, and a Keyence BZ‐X810 with a 40x objective. Pentachrome staining to visualize heart valve morphology and composition was performed with StatLab kit (NC932114). Pentachrome stained slides were imaged on an Olympus IX51 microscope with a 10x objective. For immunostaining to detect Acta2 protein, slides were incubated in 10mM sodium citrate buffer (pH 6) then blocked in blocking buffer (1% bovine serum albumin, 0.1% fish skin gelatin, 0.1% Triton X‐100 in PBS). Sections were incubated with Acta2 primary antibody (1:500, Sigma A2547) overnight at 4℃, followed by a 1‐h room temperature incubation with secondary antibody donkey anti‐mouse Alexafluor 568 (1:400, Life Technologies A10037). Slides were mounted in Vectashield Hardset Antifade mounting medium with DAPI (Vector Labs) and imaged on an Olympus IX51 microscope with a 40x objective. Image analysis was performed using ImageJ software.

### 2.4 Mean linear intercept

The mean linear intercept (MLI) was calculated to determine average distance between the alveolar walls. To calculate MLI, a 1000‐μm^2^ grid was overlaid onto 40x lung H&E images using ImageJ software. One count was made for each time a grid line crossed over an alveolar wall. Six grid lines were counted for each image, three horizontal and three vertical. The following equation was used to calculate the MLI for each image: # horizontal intercepts + # vertical intercepts/total area (µm^2^). Five images were quantified per lung and their averages were taken for the MLI of each lung.

### 2.5 Pulmonary muscularization

To assess muscularization of pulmonary vessels, 10 H&E stained blood vessels from each mouse lung ranging from 10 to 50 μm in diameter were imaged at 40x magnification. ImageJ software was used to measure the external and the internal area of the vessel. From this the average external diameter and average internal diameter were calculated using πr^2^ and the difference was taken to obtain the average width of the vascular wall for each vessel. The average of the 10 vessels from the control and the experimental mice were combined to determine significant difference. Smooth muscle cell number was determined by counting nuclei of H&E images.

### 2.6 Cell culture and mimic transfection

Human pulmonary artery smooth muscle cells (Lonza CC‐2581) were plated in a 12‐well plate at 5 × 10^4^ cells/well for 12 h. Cells were transfected with miR‐145‐3p (Sigma assay HM10225), miR‐145‐5p (Ambion assay ID: MC11480) or control RNA mimic at 40 nM using Lipofectamine RNAiMAX (Invitrogen) as per manufacturer's instructions. Forty‐eight h after transfection, cells were collected for RNA isolation and quantitative PCR. Transfection efficiency was determined to be approximately 60% based on Ambion Cy3 dye‐labeled synthetic miRNA mimic (ThermoFisher).

### 2.7 Quantitative real‐time polymerase chain reaction (qPCR)

Tissue taken from the right ventricle of the heart and from the superior lobe of the right lung was homogenized using the TissueLyzer II (Qiagen). Total RNA from tissue or from cell pellets of pulmonary artery cultured cells were extracted using the RiboZol^TM^ RNA extraction reagent according to the protocol provided by the manufacturer (VWR). RNA concentration was measured using a Nanodrop ND‐1000 (Thermofisher). cDNA was generated using 10 to 50 ng of RNA and M‐MLV reverse transcriptase (Promega). Real‐time quantitative polymerase chain reaction was prepared with Power SYBR^®^ Green Master Mix (Applied Biosystems) and performed using the StepOne polymerase chain reaction system (Applied Biosystems). Quantification was performed using the ΔΔCt method with Rpl13 used as a normalizing control. Primer sets for qPCR are listed in supplemental table.

### 2.8 LacZ staining

Harvested lung and heart tissue were fixed at room temperature for 2 h in a solution of 2% formaldehyde, 0.2% glutaraldehyde, in PBS, pH 7, then incubated overnight at room temperature in stain solution (100mM ferrocyanide/ferricyanide mix, 1 M MgCl_2_, 10mg X‐GAL dissolved in dimethylformamide, 0.1% Tween‐20 in PBS). Following the incubation, organs were fixed in 4% PFA overnight at 4℃ then cleared with a 1:2 ratio of benzyl alcohol:benzyl benzoate (Sigma) (Anderson et al., 2004). Images were captured with an Olympus SZX7 dissecting scope with a DF Plapo 1X objective at variable magnification.

### 2.9 Single cell RNA analysis

Lungs from postnatal day 12 mice were collected and washed in cold PBS. Tissue was cut into pieces and digested in 2mg/ml Collagenase Type II (Worthington Biochemical, LS004174) for 45 min at 37℃, pipette mixing was carried out to dissociate cells every 10 min. Cells were filtered through 100 and 70 µm cell strainers (BD Falcon) and treated with red blood cell lysis buffer (Qiagen) for 5 min. Cells were resuspended in 250 µl culture media (10% Fetal Bovine Serum, DMEM High Glucose with Glutamine, Penicillin Streptomycin). Cell viability (>85%) was measured using the Automated Cell Counter (Thermo Fisher, AMQAF1000). Single‐cell droplet libraries (~2000 target cell recovery/group) were generated using the Chromium Single Cell 3’ Reagent Kit v2 controller (10x Genomics) according to the manufacturer's instructions. cDNA libraries were quality checked and quantified using the High Sensitivity D5000 and D1000 ScreenTape on the 2200 TapeStation (Agilent). All libraries were sequenced (read length 150bp, pair ended) on the Illumina HiSeq4000 platform. Sequencing was performed by the Biomedical Genomics Core of The Abigail Wexner Research Institute at Nationwide Children's Hospital. The Illumina.bcl files were demultiplexed and converted into Fastq files using the cellranger `mkfastq` pipeline. The Fastq files were aligned against mouse genome version mm10 genome version index from 10X genomics (https://cf.10xgenomics.com/supp/cell‐exp/refdata‐gex‐mm10‐2020‐A.tar.gz) using `count` function of the cell ranger version 5.0 (10x Genomics) using the High Performance Computing Facility at The Abigail Wexner Research Institute at Nationwide Children's Hospital. Subsequent processing, quality control, principal component analysis, dimensionality reduction, batch‐correction, and integration were performed using the Seurat (v 3.2) package in R (Stuart & Satija, 2019). Cell types were identified using the markers identified for each cluster and compared to previously annotated clusters identified in LungMAP datasets. The results here are in part based upon data generated by the LungMAP Consortium [U01HL122642] and downloaded from LungMAP (www.lungmap.net). The LungMAP consortium and the LungMAP Data Coordinating Center (1U01HL122638) are funded by the National Heart, Lung, and Blood Institute (NHLBI).

### 2.10 Statistical analysis

All data are presented as mean ± SD. GraphPad Prism (GraphPad Software, Inc) was used for statistical analyses. Student's *t*‐test or ANOVA was performed. For single cell RNA‐seq data, Fisher exact test was used as indicated. For cell number comparisons, cells were normalized to the total number of cells analyzed. Differences were considered significant if *p *< 0.05, or as indicated. Data shown are representative of ≥5 independent experiments, or with indicated sample number.

## 3 RESULTS

### 3.1 Overexpression of *miR*‐*145* causes premature death in mice

**FIGURE 1:**
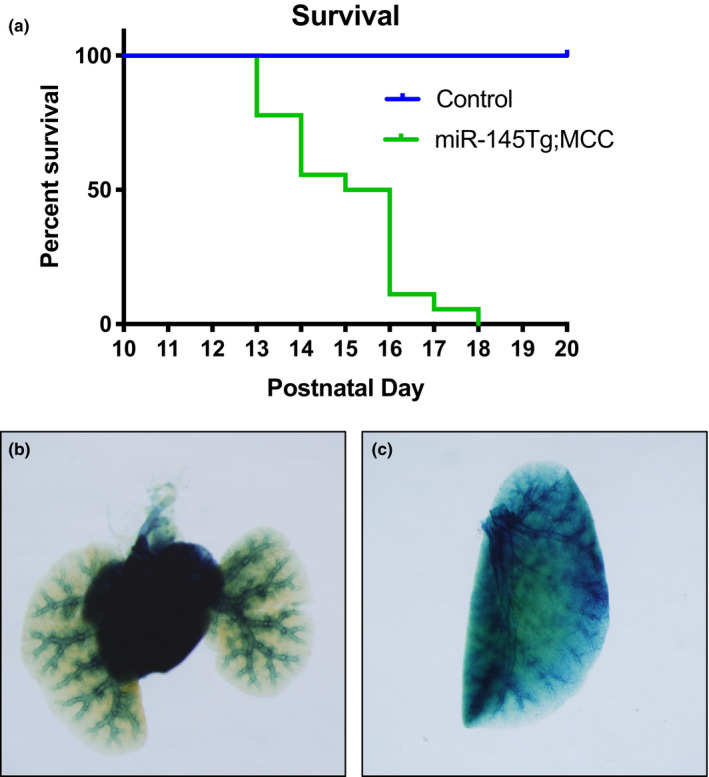
Overexpression of *miR*‐*145* in the cardiovascular system leads to premature death in mice. Kaplan–Meier survival curve to illustrate the range in which *miR*‐*145* transgenic mice *(miR*‐*145Tg*;*MCC)* succumb. Genotype of control mice were +/+;+/+, *miR*‐*145Tg*/+;+/+, or +/+;*MCC*/+ (n = 18) (a). *LacZ* expression demonstrates expression of the *Myocardin*‐*Cre* transgene *(MCC)* in the hearts and lungs of mice at embryonic day (E)16.5 (b), and in the lungs in postnatal day 14 mice (c) (n = 3)

**FIGURE 2:**
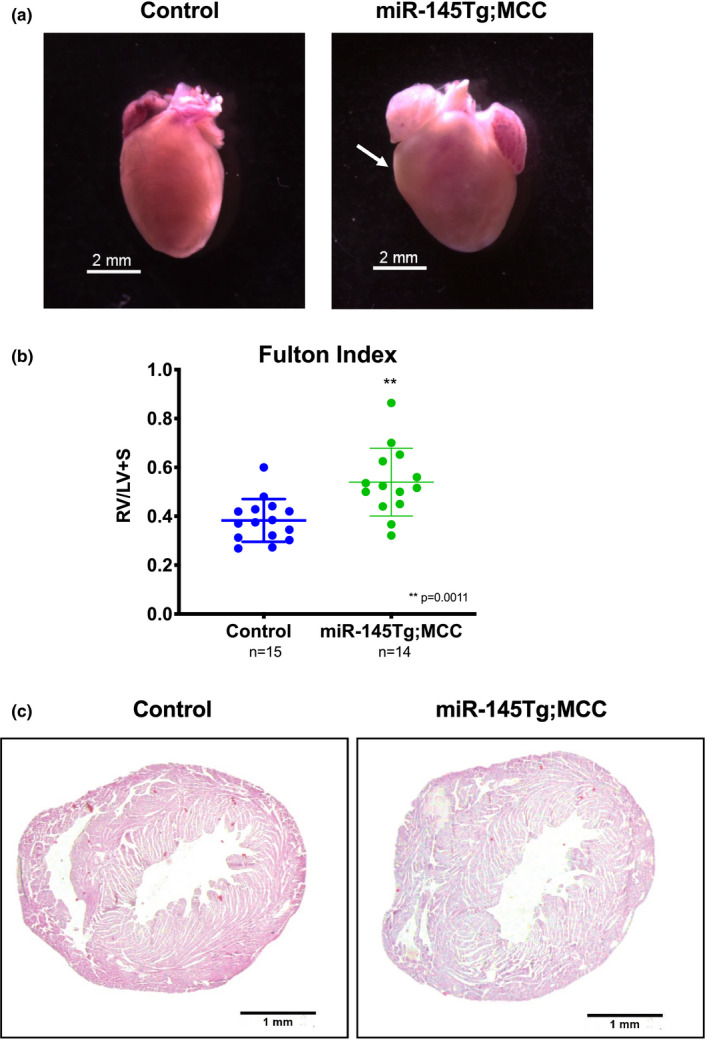
*miR*‐*145* transgenic mice exhibit right ventricular hypertrophy. Dissected hearts from control and *miR*‐*145Tg*;*MCC* mice show *miR*‐*145* overexpressing mice have an enlarged right ventricle (arrow) (n = 7) (a). Fulton index show weight ratio of right ventricle (RV) to left ventricle and septum is significantly greater in the *miR*‐*145Tg*;*MCC* hearts compared to controls (b). H&E staining of cross‐sectioned hearts demonstrate increased thickness of the RV wall in *miR*‐*145Tg*;*MCC* mice (n = 7) (c). Genotype of control mice were +/+;+/+, *miR*‐*145Tg*/+;+/+, or +/+;*MCC*/+ (n = 7)

To better define the role of *miR*‐*145* in the cardiovascular system, we utilized a transgenic mouse strain to conditionally overexpress *miR*‐*145 (miR*‐*145Tg)* through LoxP recombination (Sawant et al., 2020). We crossed the *miR*‐*145Tg* mice to a *Myocardin*‐*Cre (MCC)* driver mouse line, which drives Cre recombinase expression initially in the cardiac forming tissues at embryonic day (E)7.5 and subsequently in smooth muscle cells from E9.5 onwards (Long et al., 2007). Progeny from this cross resulted in overexpression of *miR*‐*145 (miR*‐*145Tg*;*MCC)* in a pattern that reflects its endogenous expression, beginning at E7.5 (Cordes et al., 2009). Expression was 5‐ to 10‐fold higher than endogenous levels and sustained following Cre‐mediated excision (Sawant et al., 2020). The double transgenic mice were born at expected Mendelian frequency and initially appeared normal (Sawant et al., 2020). However, the *miR*‐*145Tg*;*MCC* mice began to die at postnatal day (P)13, and no mice survived past P18, with most succumbing between P13‐16 (Figure 1a). The *miR*‐*145* transgenic mice failed to thrive and exhibited dyspnea prior to their death, suggesting potential heart and/or lung abnormalities. We confirmed *Myocardin*‐*Cre* expression in the hearts and lungs by crossing with the *Rosa26*‐*LacZ (Gt(ROSA)26Sor^tm1Sor^)* reporter mouse strain, (Soriano, 1999) which showed expression of Cre primarily in the heart and pulmonary vasculature, embryonically as well as postnatally (Figure 1b, c). To further investigate the cause of death, we analyzed the heart and lungs of mice at P14, a time point at which the pups were noticeably frailer than their littermates. Analysis of the hearts at P14 revealed an enlarged right ventricle in the *miR*‐*145Tg*;*MCC* mice compared to control mice (Figure 2a). Quantification of this enlargement by Fulton index to measure the right to left ventricle ratio, showed a significant hypertrophy compared to control mice (Figure 2b). Total heart weight was not significantly different between the groups (not shown). Transverse sections followed by H&E staining through the hearts, revealed a thickened right ventricle wall in the *miR*‐*145* overexpressing mice (Figure 2c). We analyzed hearts of mice at P2 to assess if the right ventricular hypertrophy was present soon after birth. At P2 the *miR*‐*145Tg*;*MCC* hearts were indistinguishable from control hearts and did not exhibit right‐sided enlargement based on Fulton index (Supplementary Figure S1a, b). Together, these results demonstrate that the *miR*‐*145Tg*;*MCC* pups display secondary right ventricular hypertrophy, which is present at P14 and beyond prior to their death before P18.

### 3.2 *miR*‐*145* transgenic mice show cardiac abnormalities

**FIGURE 3:**
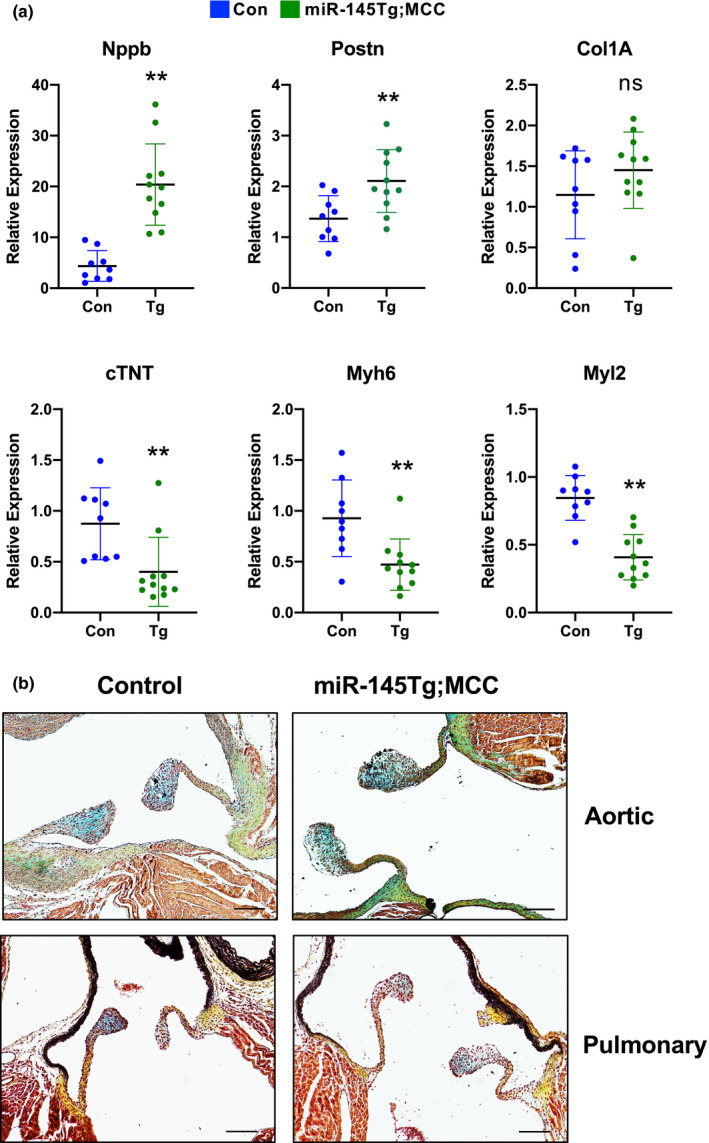
Hearts of *miR*‐*145Tg*;*MCC* mice show evidence of heart injury. Increased mRNA expression of *BNP (Nppb)*, *Postn*, and *Col1A* in transgenic mice suggest cardiac injury, with a concomitant decrease in cardiac markers, *cTNT (Tnnt2)*, *Myh6*, and *Myl2*, in *miR*‐*145Tg*;*MCC* (Tg, n = 11) hearts compared to control (Con, n=9) hearts (a). ** *p *< 0.05, ns =not significant. Histological analysis using pentachrome staining of the aortic and pulmonary valves indicates no obvious defects in the valves of the transgenic mice, (n = 6) scale bar = 100µm (b). Genotype of control mice were +/+;+/+, *miR*‐*145Tg*/+;+/+, or +/+;*MCC*/+

The *miR*‐*145Tg*;*MCC* mice showed right ventricle hypertrophy at P14, a timepoint in which the double transgenic mice began to die, suggesting cardiac abnormalities. To investigate further, we measured markers of cardiac injury by quantitative reverse transcriptase PCR and the data show that expression of known heart failure and fibrosis markers *BNP (Nppb)*, *periostin (postn)*, and *Collagen 1A (Col1A)* mRNA are all elevated in the right ventricles of the transgenic mice compared to control (Figure 3a). Analysis of cardiomyocyte‐restricted markers *cTNT (Tnnt2)*, *Myh6*, and *Myl2* showed a significant reduction in the *miR*‐*145Tg*;*MCC* mice compared to control mice (Figure 3a). To determine if the heart abnormalities were a potential secondary effect due to heart valve deformities, we examined the valves of the heart with pentachrome stain at P14. The aortic and pulmonary valves of the *miR*‐*145Tg*;*MCC* hearts appeared normal (Figure 3b), as did the mitral and triscuspid valves (not shown). Compared to control valves, *miR*‐*145Tg*;*MCC* valves appeared structurally normal and did not show evidence of an altered cellular or extracellular composition, suggesting that the cardiac abnormalities were not due to defects of the valves.

### 3.3 Lungs of *miR*‐*145* transgenic mice are dysplastic and show vascular hypertrophy

**FIGURE 4:**
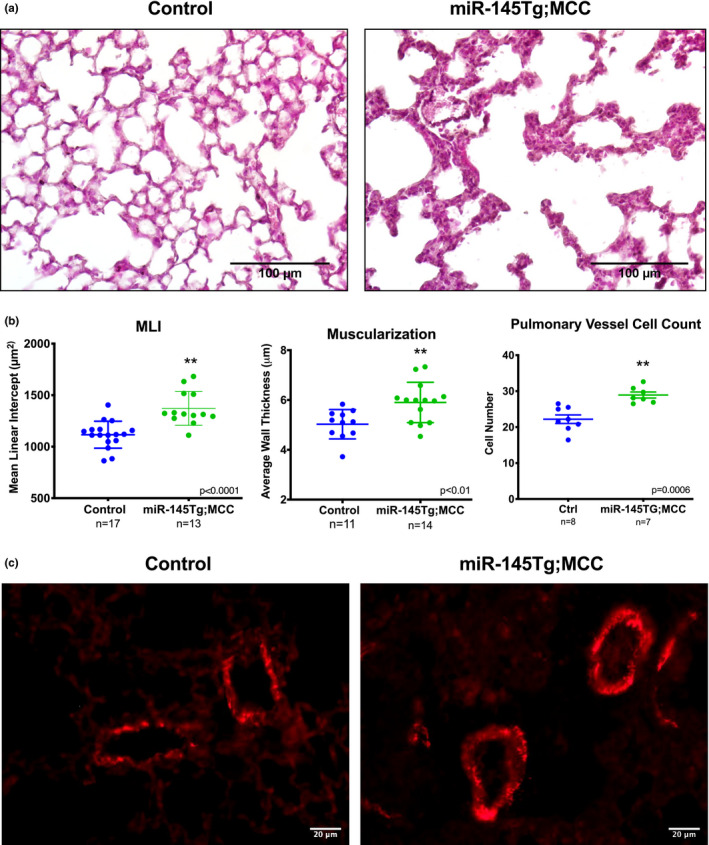
Lungs of *miR*‐*145Tg*;*MCC* mice exhibit alveolar remodeling and increased vessel muscularization. H&E staining of lung sections of control and miR‐145 transgenic mice show alterations in lung architecture and alveolar remodeling (a) (n = 10). Quantification of structural differences by mean linear intercept (MLI) indicates transgenic mice are significantly different from control mice (b). Degree of blood vessel muscularization was determined by measuring wall thickness (c), counting cell number (d), and staining for smooth muscle α‐actin (Acta2) (e), which shows an increase in smooth muscle surrounding small caliber arteries in the lung (n = 6)

**FIGURE 5:**
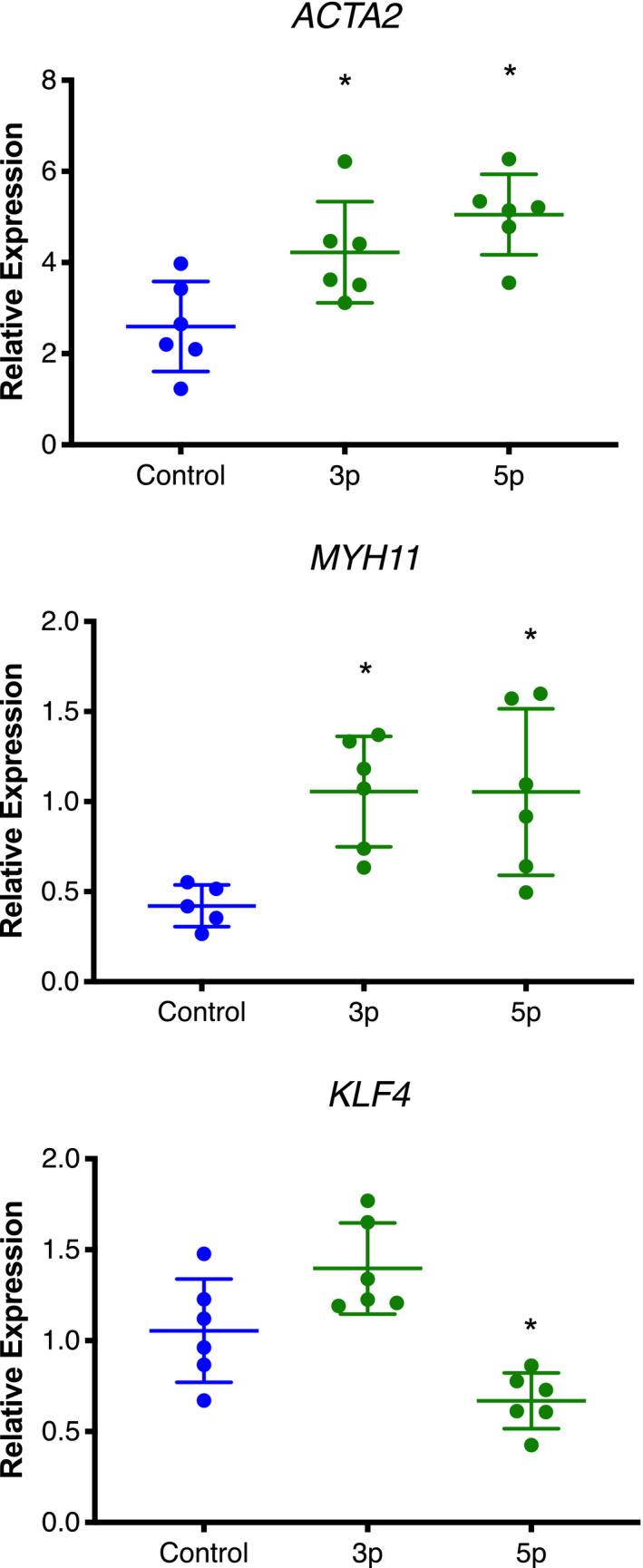
*miR*‐*145* induces the expression of smooth muscle marker genes in pulmonary artery smooth muscle cells. Human pulmonary artery smooth muscle cells (hPASMCs) were transfected with control (Con), *miR*‐*145*‐*3p* (3p), or *miR*‐*145*‐*5p* (5p) mimics followed by RNA isolation and qPCR to detect marker gene expression as indicated (n = 6). *p < 0.05, relative to control

The *miR*‐*145* transgenic mice exhibited dyspnea prior to death and dissection revealed a hypertrophied right ventricle, indicative of pulmonary dysplasia and/or hypertension. Examination of the pulmonary structure by H&E staining of lung sections of P14 mice revealed significant differences in the alveolar networks, indicative of pulmonary dysplasia (Figure 4a) (Ahlfeld et al., 2013, 2016). These differences were quantified using the mean linear intercept (MLI), which revealed that the *miR*‐*145Tg*;*MCC* mice had lungs with significantly altered alveolar network (Figure 4b). Evaluation of lung architecture at P2 revealed no significant differences between control and *miR*‐*145* transgenic lungs (Supplementary Figure S1c, d), indicating the abnormalities were acquired after birth, following inflation of fluid filled fetal lungs and after the increase in blood flow resistance. To assess whether the vasculature was hypertrophied, indicative of pulmonary hypertension, we examined vessel wall thickness, and stained lung sections of P14 mice to detect protein expression of smooth muscle α‐actin (Acta2) and measured vessel wall diameter (Figure 4c‐e). Our data show that the *miR*‐*145* overexpressing mice had increased wall thickness and smooth muscle cell number (Figure 4c, d), and increased Acta2 surrounding small caliber vessels (Figure 4e). Analysis of *Acta2* mRNA expression in lung extracts of control and *miR*‐*145Tg*;*MCC* did not reveal significant differences (not shown). To determine if *miR*‐*145* could induce *Acta2* expression in cultured smooth muscle cells, we transfected human pulmonary artery smooth muscle cells (hPASMCs) with *miR*‐*145* mimics. Introduction of *miR*‐*145*‐*3p* and *miR*‐*145*‐*5p* into hPASMCs revealed that both *miR*‐*145* mimics could induce *ACTA2* mRNA expression, as well as *smooth muscle myosin heavy chain* (*MYH11*) expression (Figure 5). In contrast, and as expected, known target *KLF4* was downregulated by *miR*‐*145*‐*5p* (Figure 5). These data indicate that *miR*‐*145* transgene expression is driving excess muscularization in the lungs of transgenic pups, which likely contributes to the phenotypic consequences observed in the *miR*‐*145Tg*;*MCC* mice.

### 3.4 Single cell sequencing reveals pathways that are uniquely regulated by *miR*‐*145*

**FIGURE 6:**
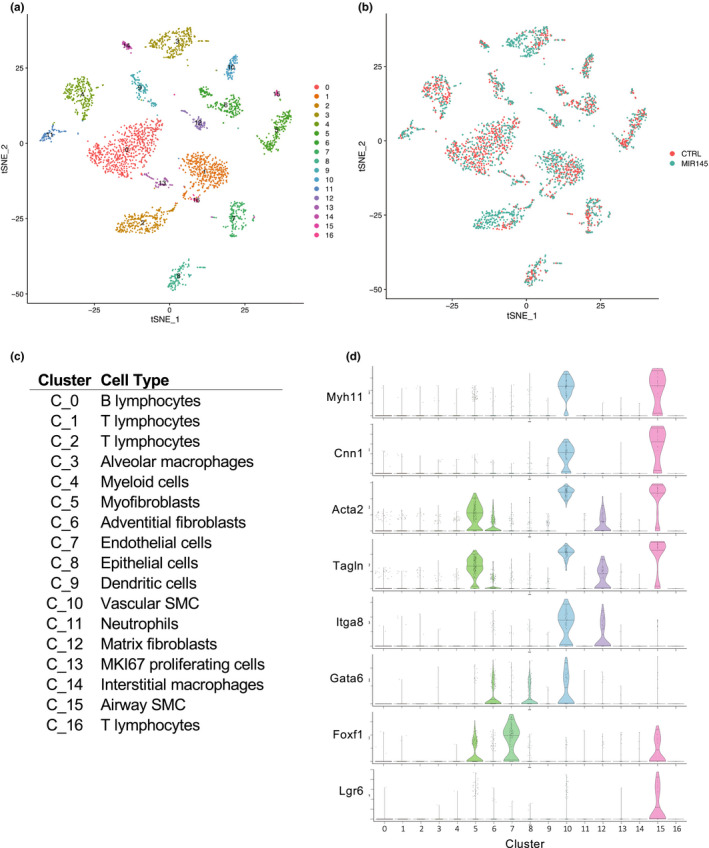
Single cell RNA‐seq of wild‐type and *miR*‐*145Tg* P13 lungs. t‐SNE plot of cell clusters from wild‐type and *miR*‐*145*‐*Tg*;*MCC* lungs combined (a). Overlap of cells from wild‐type (CTRL) and *miR*‐*145*‐*Tg*;*MCC* (MIR145) lungs (b). Cell type identification of clusters (c) and violin plots of smooth muscle marker genes reveals vascular (cluster 10) and airway (cluster 15) smooth muscle cell subpopulations (d)

To gain a better understanding of how overexpression of *miR*‐*145* was affecting the lungs, we performed single‐cell mRNA sequencing analysis on whole lung tissue from P13 wild‐type and *miR*‐*145Tg*;*MCC* mice. The analyses utilized sequence data from 1600 wild‐type cells and 1775 *miR*‐*145Tg*;*MCC* cells. t‐SNE plot (Figure 6a) and heatmap (Supplemental Figure S2) show 17 clusters identified using unsupervised clustering method in Seurat, (Stuart & Satija, 2019) with the wild‐type and *miR*‐*145Tg* cell groups uniformly overlapping in different clusters (Figure 6b). Cluster cell types were identified based on classification using the LungMAP single cell RNA‐seq *Mus musculus* P14 database (www.lungmap.net), Mouse Cell Atlas (http://bis.zju.edu.cn/MCA/), and relevant single‐cell RNA‐seq published datasets (Figure 6c) (Peyser et al., 2019; Travaglini et al., 2020; Tsukui et al., 2020; Xie et al., 2018). Immune cell subpopulations made up 9 of the 17 clusters. Based on *Myocd*‐*Cre* expression (Figure 1b, c), the *miR*‐*145Tg* is primarily expressed in the smooth muscle population (Long et al., 2007). Five clusters (C) (C5, C6, C10, C12, and C15) were identified as being myocyte/fibroblast‐like, based on expression of differentiation‐specific smooth muscle markers, *Myh11* and *Cnn1*, and shared fibroblast markers *Acta2* and *Tagln* (Figure 6d). Clusters C10 and C15 were identified as vascular smooth muscle (VSMC) and airway smooth muscle (ASMC), respectively, based on additional specific markers, *Itga8* and *Gata6* for VSMC, and *Foxf1* and *Lgr6* for ASMC (Figure 6d) (Kitchen et al., 2013; Lee et al., 2017; Morrisey et al., 1996; Ustiyan et al., 2018). The remaining myocyte/fibroblast‐like clusters were further identified as myofibroblasts (C5), adventitial fibroblasts (C6), and matrix fibroblasts (C12) (Supplementary Figure S3).

**FIGURE 7:**
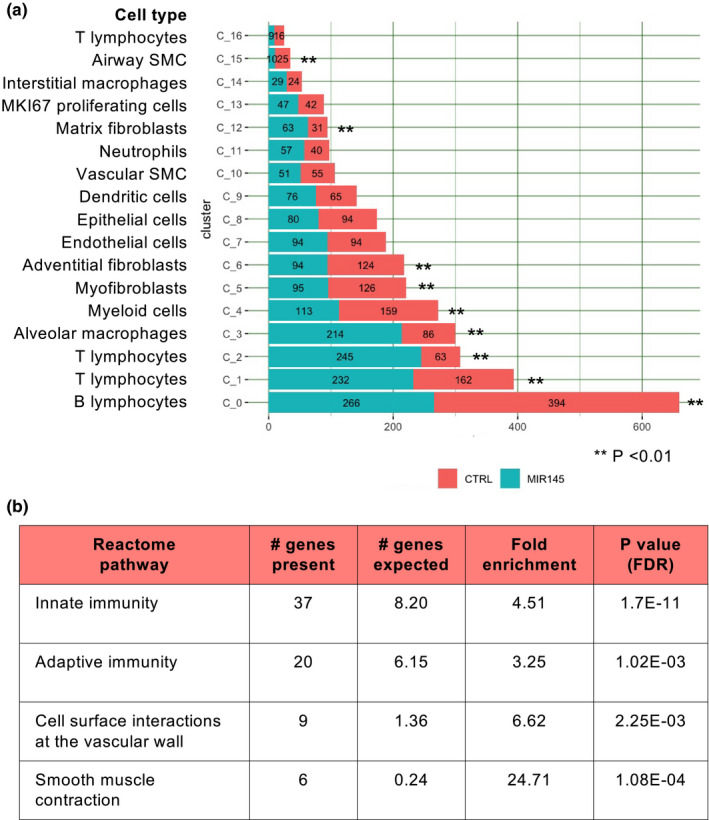
miR‐145Tg alters cell number and cell cycle of distinct cell types. Graph of cell number in each cluster from wild‐type (CTRL) and miR‐145Tg (MIR145) P13 lungs (a). Significance P value determined by Fisher exact test. Reactome pathway table identified by differential gene expression shows pathways that are most affected by overexpression of miR‐145Tg (b). FDR (false discovery rate)

Cell number comparisons from the single‐cell dataset revealed that immune cells and fibroblast populations were significantly different between wild‐type and *miR*‐*145Tg*;*MCC* lungs (Figure 7a). T lymphocytes (C1 and C2) were increased greater than two‐fold in the *miR*‐*145* transgenic lungs and the myeloid cell population, specifically alveolar macrophages (C3 and C4), were also increased in the *miR*‐*145Tg*;*MCC* compared to control. In contrast, the B lymphocyte population (C0) was greater in wild‐type lungs compared to transgenic mouse lungs. In the myocyte/fibroblasts clusters, adventitial fibroblasts (C6), myofibroblasts (C5), and airway smooth muscle cells (C15) had reduced cell numbers in the *miR*‐*145Tg*;*MCC* compared to control, while C12, the matrix fibroblasts showed an increase in cell number in the transgenic mice (Figure 7a). Cluster 10, the VSMC population was not significantly different in cell number. These data indicate the *miR*‐*145* overexpression was inducing a robust immune response in the lung with an increase in T‐cell and macrophage cell populations.

We used gene ontology enrichment analysis to examine gene expression changes in cells from wild‐type and *miR*‐*145Tg*;*MCC* lungs (Figure 7b, and supplementary Figure S4). Genes that showed the greatest differences were overwhelmingly from the innate and adaptive immunity pathways. Many of these genes were upregulated in the *miR*‐*145Tg*;*MCC* cells compared to wild‐type cells, suggesting the *miR*‐*145* is invoking a proinflammatory response within the lung. Smooth muscle contractile genes were also affected, and surprisingly this gene group showed a downregulation in the *miR*‐*145* overexpressing cells. Additionally, genes that were over‐represented fell into a category classified as “cell surface interactions at the vascular wall,” which included genes known to be involved in leukocyte extravasation, a process of transendothelial migration from the vascular lumen to sites of tissue inflammation (Weber et al., 2007). These data indicate that *miR*‐*145* is having both localized and systemic effects on gene regulation in lung tissue. Overall, the combined data indicate that *miR*‐*145* overexpression promotes a robust immune response in the lung that results in the pathological consequences leading to cardiopulmonary complications and early postnatal death.

## 4 DISCUSSION

The role of *miR*‐*145* in the cardiopulmonary system has been examined extensively, with most studies utilizing loss of function or acute delivery of *miR*‐*145* mimics or inhibitors postnally (Jakob & Landmesser, 2012; Vacante et al., 2019). Here we characterize the phenotype of transgenic mice that overexpress *miR*‐*145* in the in utero cardiopulmonary system using Cre‐mediated expression. Utilization of the *Myocardin*‐*Cre* driver to induce transgene expression revealed that overexpression of *miR*‐*145* caused postnatal lethality. Transgenic mice were born at expected Mendelian ratios, (Sawant et al., 2020) however, between postnatal day 13 and 18, pups exhibited signs of dyspnea and abruptly died. Our data show that the *miR*‐*145Tg*;*MCC* pups had phenotypic indicators of pulmonary hypertension and dysplasia, with evidence of a hypertrophied right ventricle and muscularization of the small pulmonary arteries, and abnormal remodeling of the alveolar networks. Attempts to perform right ventricular arterial pressure measurements on these fragile pups were unsuccessful, therefore it is not known if these pups were in fact hypertensive. Evidence of a pulmonary hypertensive‐like phenotype is corroborated by previous findings. Caruso et al., showed in a hypoxia‐induced pulmonary hypertension model that *miR*‐*145*‐deficient mice were protected from severe disease (Caruso et al., 2012). Our cell culture data using *miR*‐*145* mimics, confirmed that *miR*‐*145*‐*3p* and *miR*‐*145*‐*5p* could induce expression of *Acta2* and *Myh11* in pulmonary artery smooth muscle cells, consistent with *miR*‐*145* promoting differentiation and muscularization. This finding is similarly consistent with previous reports demonstrating the ability of *miR*‐*145* to induce smooth muscle gene expression (Albinsson et al., 2010; Cordes et al., 2009). Detailed examination of the hearts of the transgenic pups show significant right‐sided hypertrophy, however, the heart valves and cardiomyocytes in both ventricles appeared structurally normal. A recent report of transgenic *miR*‐*143*/*145* expression in hearts utilizing *αMHC*‐*Cre* showed left ventricular hypertrophy leading to death beginning at 8 weeks of age (Ogawa et al., 2020). Thus, the most likely cause of death of our *miR145Tg*;*MCC* pups is in response to pulmonary distress caused by pathological pulmonary remodeling. Whether this is a direct effect of *miR*‐*145* on the smooth muscle lineage is not clear. Possibly, *miR*‐*145* overexpression alters normal vascular muscularization that causes a proinflammatory response leading to disease pathology.

To address the global effect that *miR*‐*145* overexpression had on lung tissue, we performed single cell RNA‐sequencing of control and *miR*‐*145Tg*;*MCC* lungs. The data show that *miR*‐*145* overexpression has wide‐reaching effects on gene expression and cellular makeup within lung tissue. We observed an increase in the number of T lymphocytes and myeloid cells, and decreased number of B lymphocytes. It is well established that inflammation plays a role in pulmonary hypertension, however, whether it is an upstream driver or consequence is still debated (Huertas et al., 2020; Rabinovitch et al., 2014). A maladapted immune response is present in pulmonary hypertension, and this results in the accumulation of perivascular inflammatory cells and the overabundance of cytokines and chemokines (Rabinovitch et al., 2014). Studies have demonstrated that localized vascular inflammation, involving the endothelium, smooth muscle cells, and the adventitia can induce a systemic inflammatory response that contributes to disease progression (Hu et al., 2020; Huertas et al., 2020). Consistent with this, examination of differential gene analysis between the *miR*‐*145Tg*;*MCC* lungs and controls showed that genes in the innate and adaptive immunity pathways were the most affected and indicates a robust inflammatory reaction in the *miR*‐*145Tg* lungs. Further, genes classified in the “cell surface interactions at the vascular wall,” pathway were also affected, which comprise genes that contribute to leukocytes traversing the endothelium in response to tissue inflammation. Together, these results imply that overexpression of *miR*‐*145* in VSMC is contributing to systemic disease.

Smooth muscle contractile genes also exhibited differential expression, but surprisingly these data show that the genes were collectively downregulated in the *miR*‐*145Tg*;*MCC* lungs. The decreased expression of smooth muscle markers is in direct contrast to previous studies and our own cell culture results, where *miR*‐*145* is shown to induce expression of smooth muscle marker genes. These results could reflect the advancement of lung disease that is induced by *miR*‐*145* overexpression and therefore could be consequential rather than causative. Furthermore, the ability of *miR*‐*145* to increase expression of smooth muscle marker genes is known to be indirect, and therefore in the *miR*‐*145Tg*;*MCC* lung samples this pathway may be dysfunctional. While our results cannot be fully explained, they suggest that *miR*‐*145* function is largely context dependent, and generalized conclusions must be carefully considered. Overall, these data demonstrate that *miR*‐*145* plays a critical role in homeostasis and disease of the cardiovascular system, and its expression levels can have a major impact that extends beyond the regulation of smooth muscle cell function.

## DISCLOSURES

The authors have declared no conflicts of interest.

## AUTHORS CONTRIBUTIONS

ST performed the experiments, collected, and analyzed data, processed data with statistical analysis. SM, DS, and KK performed the experiments, collected, and analyzed data with statistical analysis. VG and SC helped design experiments, reviewed data with statistical analysis, and provided edits to the manuscript. BL designed the experiments, performed experiments, processed data with statistical analysis and wrote the manuscript. All authors reviewed and revised the manuscript, approved the final manuscript as submitted, and agree to be accountable for all aspects of the work.

## Supporting information



Supplementary Material

Supplementary Material
