# Supplementary material for: miR‐145 transgenic mice develop cardiopulmonary complications leading to postnatal death
Source: Physiol Rep. 2021 Sep 14;9(17):e15013. doi: 10.14814/phy2.15013 (PMC8440944; doi:10.14814/phy2.15013)
Supplement: Supplementary file 1 — Supplementary Material [file PHY2-9-e15013-s002.pdf]

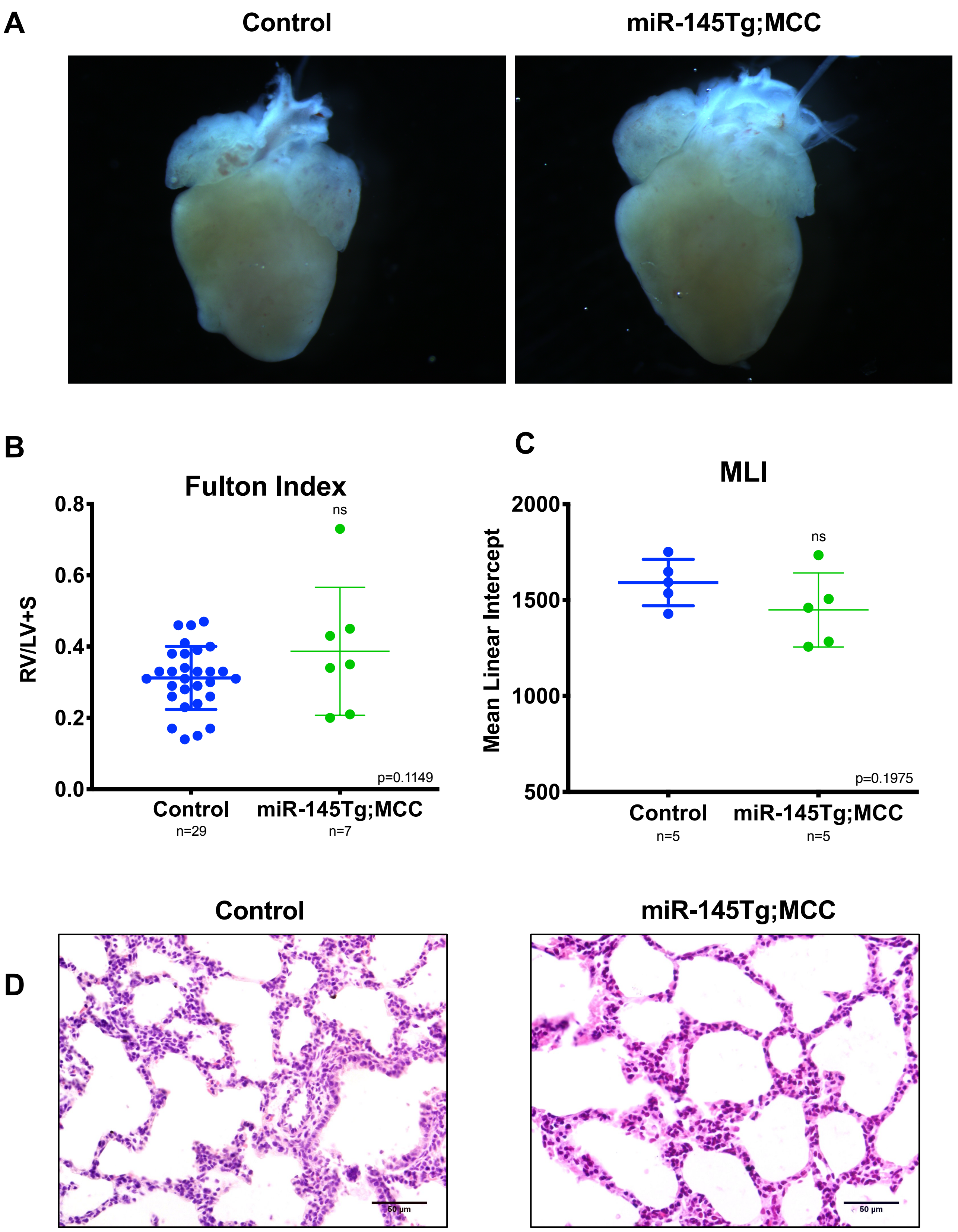

Supplementary Figure 1. Hearts and lungs from P2 mice. Images of hearts isolated from P2 control and *miR-145Tg;MCC* mice show no obvious differences (n=7) (A). Fulton index (B). Images of lungs and quantification of pulmonary architecture by mean linear intercept (MLI) (C, D), shows no significant (ns) differences between control and *miR-145Tg;MCC* lungs (n=7).

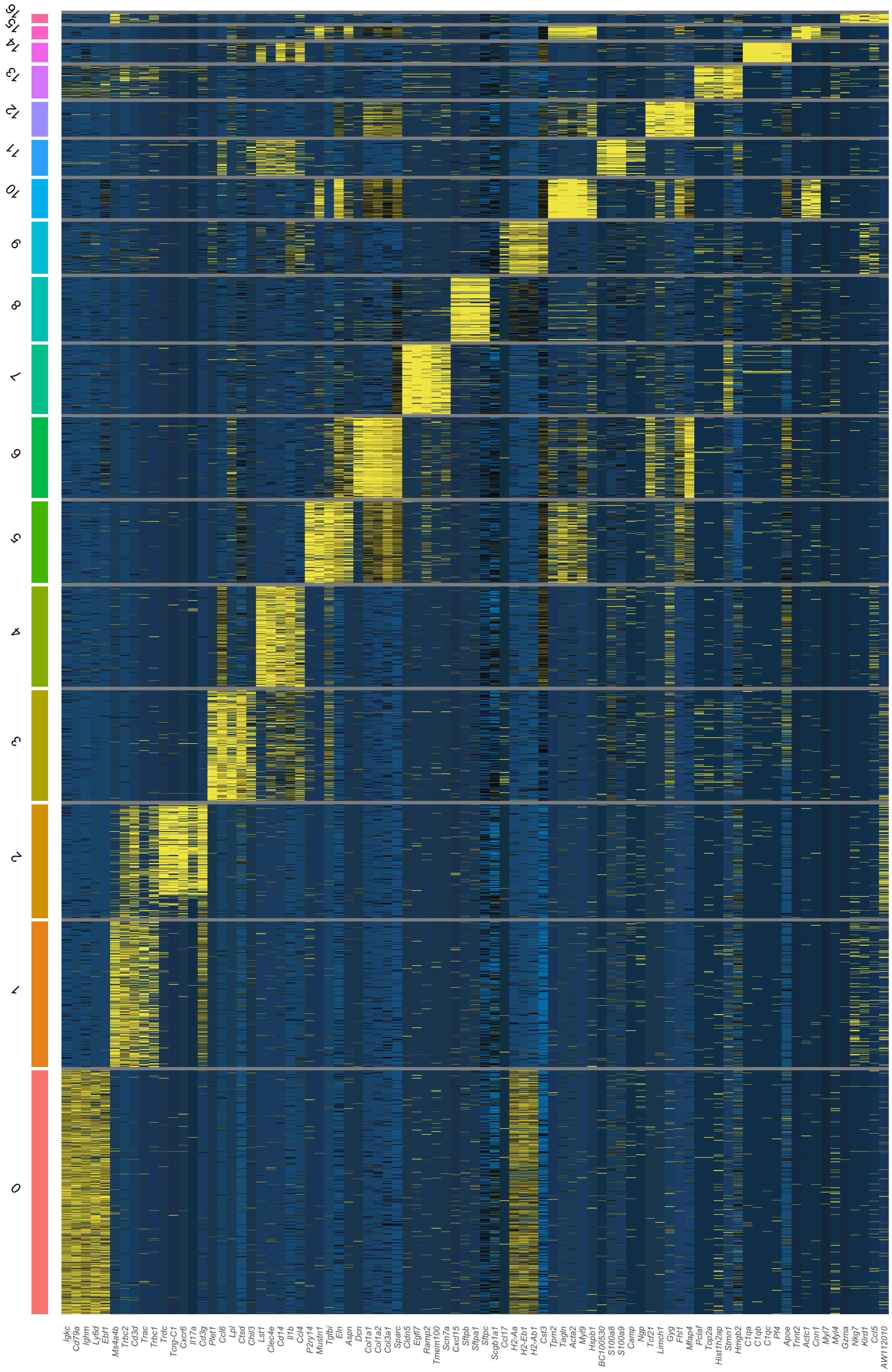

Supplementary Figure 2. Heat map of single cell RNA-seq clusters 0-16.

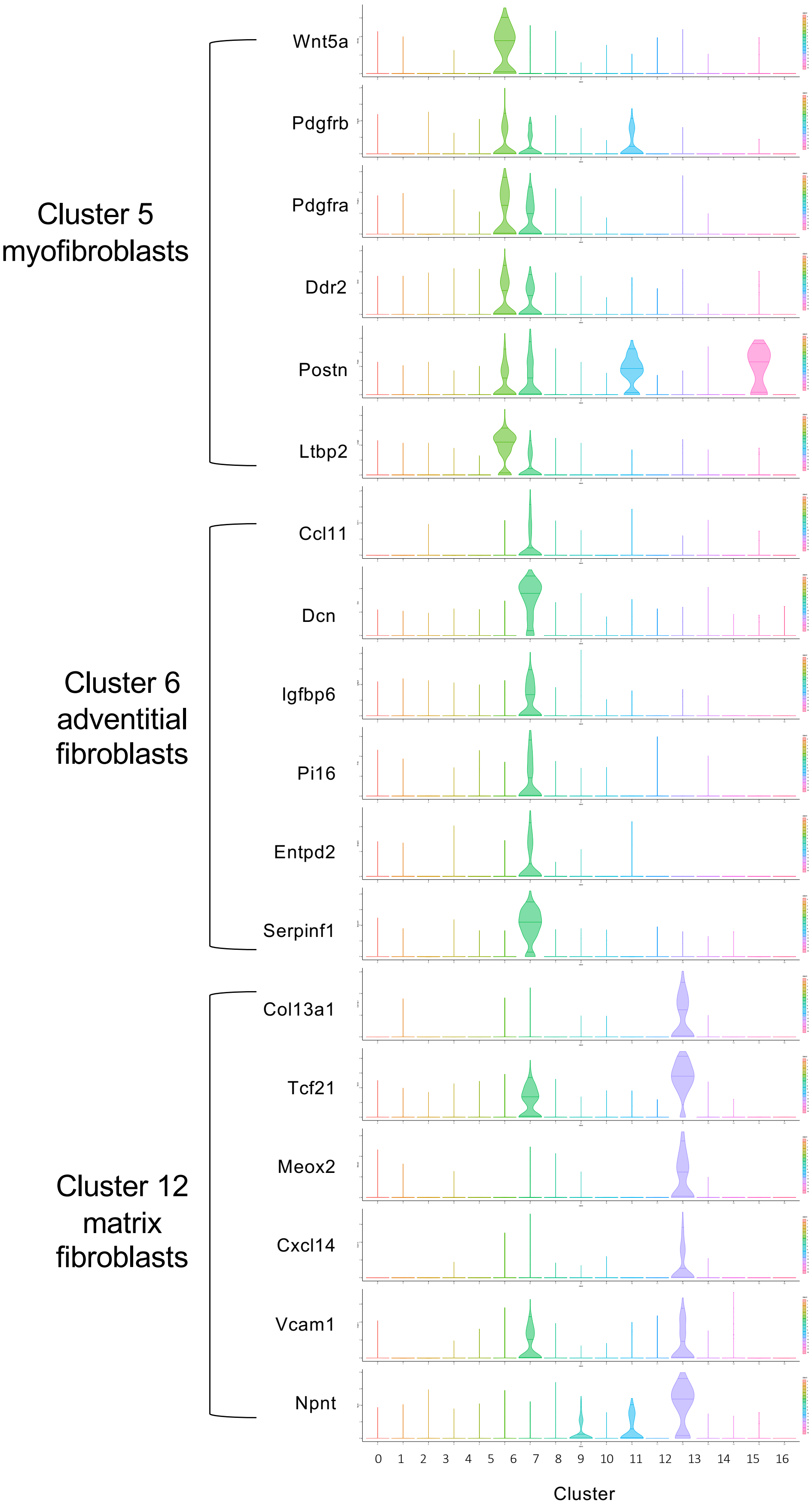

Supplementary Figure 3. Violin plots of fibroblast marker genes to identify unique subpopulations of of clusters 5, 6, and 12.

Innate immunity

Gene Name	Log2 FC	Adjusted P value
Ltf	2.878738816	2.71E-05
Camp	2.680196751	3.33E-05
Trem2	2.234609687	4.68E-12
Cd3g	1.99352269	2.61E-54
S100a8	1.942945447	7.23E-07
Syngr1	1.873532088	2.71E-05
Cxcl3	1.834971108	3.69E-08
S100a9	1.701317454	1.49E-05
Atp6v0d2	1.661255794	2.31E-10
Ctsd	1.592088709	3.37E-48
Cd247	1.549950614	7.69E-08
Lat	1.50381165	2.10E-12
Cxcl1	1.395379585	1.43E-08
Clec4n	1.393994244	2.18E-09
Bst1	1.383442973	7.13E-05
Lcn2	1.29533987	0.007572358
Pld3	1.061891329	4.82E-08
Serpinb1a	1.033055638	0.000749588
Ctss	1.007659976	2.27E-25
Itgb2	0.990313213	9.56E-08
Ctsk	0.952533668	0.002109768
Psap	0.930237651	1.95E-26
Dgat1	0.89754451	0.0106237
Itgax	0.857906656	0.030934553
Fcgr3	0.844761405	0.000806766
Clec7a	0.842254434	2.75E-07
Fth1	0.838095189	5.80E-23
Glipr1	0.822123918	0.001073216
Creg1	0.801020842	1.31E-06
Cd68	0.729688181	0.000342277
Vat1	0.717327798	0.010071625
Asah1	0.65294042	0.00134038
Lgmn	0.630838129	0.000185182
Cstb	0.61078999	4.70E-08
Plac8	-0.914435868	3.95E-13
Ms4a1	-1.078936733	6.66E-08

Adaptive immunity

Gene Name	Log2 FC	Adjusted P value
Pdcd1	3.349896145	3.71E-14
Trem2	2.234609687	4.68E-12
Icos	2.163879277	2.70E-16
Cd3g	1.99352269	2.61E-54
Zap70	1.961715025	3.87E-06
Ctsd	1.592088709	3.37E-48
Cd247	1.549950614	7.69E-08
Lat	1.50381165	2.10E-12
Cd3e	1.486150541	9.79E-13
Znrf1	1.041557456	3.21E-05
Ctss	1.007659976	2.27E-25
Itgb2	0.990313213	9.56E-08
Ctsk	0.952533668	0.002109768
Cd8b1	0.879453788	0.022996314
Lgmn	0.630838129	0.000185182
Ighm	-0.592177755	2.81E-05
Col1a2	-0.704767324	3.83E-19
Cd79a	-0.964013322	1.06E-11
Cd79b	-1.045480504	1.51E-11
Fbxl22	-1.148076609	0.003542624

SMC contraction

Gene Name	Log2 FC	Adjusted P value
Myl7	-4.203453229	0.00151146
Tpm2	-1.043338287	0.001656691
Acta2	-0.834821453	0.015181788
Myh11	-0.819308903	0.011698636
Myl9	-0.688938245	6.99E-05
Mylk	-0.613988128	0.014704108

Cell surface interactions - vascular wall

Gene Name	Log2 FC	Adjusted P value
Itgb2	0.990313213	9.56E-08
Itgax	0.857906656	0.030934553
Slc7a11	0.760314193	7.23E-07
Ighm	-0.592177755	2.81E-05
Col1a2	-0.704767324	3.83E-19
Vpreb3	-1.025022329	0.000469144
Iglc2	-1.183066082	0.005164605
Iglc3	-1.359807007	4.68E-12
Iglc1	-1.785806085	1.13E-05

Supplementary Figure 4. Gene lists from Panther Pathway Reactome analysis that are differentially expressed. (+) Log2 Fold Change (FC) indicates expression is higher in miR-145Tg cells compared to wild-type cells and (-) Log2 FC indicated expression is lower.

Primers used for qPCR of mRNA from mouse tissue

Gene Symbol<br>Forward (F) Reverse (R)	Sequence 5’-3’
Nppb-F	ACC ACC TTT GAA GTG ATC CTA TT
Nppb-R	GCA AGT TTG TGC TCC AAG ATA AG
Postn-F	TGC AAA TGC CAA CAG TTA CTA TGA
Postn-R	TGA TCC CGA CCC CTG ATG
Col1A-F	CTT CAC CTA CAG CAC CCT TGT G
Col1A-R	TGA CTG TCT TGC CCC AAG TTC
Tnnt2-F	GGA GAG AGA GTG GAC TTT GAT G
Tnnt2-R	CTT CCT CCT TCT TCC TGTTC TC
Myh6-F	CCA CCC AAG TTC GAC AAG AT
Myh-6-R	AGA AGA GGC CTG AGT AGG TAT AG
Myl2-F	GGT CAC TGA AGG CTG ACT ATG
Myl2-R	CAT CTG GTC GAT CTC CTC TTT G
Rpl13-F	CCC TCC ACC CTA TGA CAA GA
Rpl13-R	GGT ACT TCC ACC CG ACCT C

Primers used for qPCR of mRNA from human PASMC

Gene Symbol<br>Forward (F) Reverse (R)	Sequence 5’-3’
RPL13a-F	CTT GTG AGT GGG GCA TCT G
RPL13a-R	CCC TGT GTA CAA CAG CAA GC
KLF-4-F	ACCAGGCACTACCGTAAACACA
KLF4-R	ATG CTC GGT CGC ATT TTT G
MYH11-F	ACA ACC TGA GGG AGC GGT ACT
MYH11-R	CAC GCA GAA GAG GCC AGA GT
ACTA2-F	CAA GTG ATC ACC ATC GGA AAT G
ACTA2-R	GAC TCC ATC CCG ATG AAG GA
